# Undescended testis: an epidemiological study from a single institution

**DOI:** 10.1590/1984-0462/2025/43/2024186

**Published:** 2025-06-27

**Authors:** Marcela Almeida Bazaga, Márcia Emília Francisco Shida, Mila Torii Corrêa Leite

**Affiliations:** a Universidade Federal de São Paulo, Escola Paulista de Medicina – São Paulo (SP), Brazil.

**Keywords:** Cryptorchidism, Testicule, Child, Surgery, Criptorquidia, Testículo, Criança, Cirurgia

## Abstract

**Objective:**

The aim of this study was to assess the referral and treatment timelines for patients with undescended testis treated in the Pediatric Surgery Division at Escola Paulista de Medicina/UNIFESP.

**Methods:**

Retrospective review of patients treated for undescended testis at a tertiary care institution from 1992 to 2021. Ethical approval was granted by the institutional ethics committee. Data collected included patients’ demographics and clinical characteristics, referral age, age at orchidopexy, testis laterality and position prior to surgery, and duration of postoperative follow-up. A descriptive analysis was performed for each variable, excluding patients with incomplete medical records.

**Results:**

In the cohort of 350 patients with undescended testis, 72.6% had unilateral and 27.4% bilateral presentations; and 8% of the patients had differences in sex development. Ultrasound was requested in 19.5% of cases. The average age at referral was 47.3 standard deviation (±) 41.6 months, with 72.5% referred after 18 months. Among 131 orchidopexy procedures, 12.8% were performed before 12 months of age, 15.9% until 18 months, and the majority after 60 months. Of the 120 patients who underwent surgery, only 78.3% had postoperative follow-up data, and 52.1% lost follow-up. The average follow-up period was 23.1±26.20 months.

**Conclusions:**

The average age at diagnosis and treatment of undescended testis was higher than recommended by international guidelines, indicating the need for enhanced medical and non-medical community referral practices.

## RESUMO

**Objetivo:**

O objetivo deste estudo foi avaliar os tempos de encaminhamento e tratamento dos pacientes com testículos não descidos atendidos na Disciplina de Cirurgia Pediátrica da Escola Paulista de Medicina/UNIFESP.

**Métodos:**

Revisão retrospectiva dos pacientes tratados para testículos não descidos em uma instituição de cuidado terciário de 1992 a 2021. A aprovação ética foi concedida pelo comitê de ética institucional. Os dados coletados incluíram características demográficas e clínicas dos pacientes, idade de encaminhamento, idade na época da orquidopexia, lateralidade e posição dos testículos antes da cirurgia, e duração do acompanhamento pós-operatório. Foi realizada uma análise descritiva de cada variável, excluindo os pacientes com registros médicos incompletos.

**Resultados:**

Na coorte de 350 pacientes com testículos não descidos, 72,6% tiveram apresentações unilaterais e 27,4% bilaterais; e 8% dos pacientes apresentaram diferenças no desenvolvimento sexual. Ultrassonografia foi solicitada em 19,5% dos casos. A idade média de encaminhamento foi de 47,3 desvio padrão (±) 41,6 meses, sendo 72,5% encaminhados após 18 meses. Entre os 131 procedimentos de orquidopexia, 12,8% foram realizados antes da idade de 12 meses, 15,9% até os 18 meses e a maioria após 60 meses. Dos 120 pacientes operados, apenas 78,3% tiveram dados de acompanhamento pós-operatório, com 52,1% perdidos no acompanhamento. O período médio de acompanhamento pós-operatório foi de 23,1±26,2 meses.

**Conclusões:**

A idade média no diagnóstico e tratamento do testículo não descido foi superior ao recomendado pelas diretrizes internacionais, indicando a necessidade de melhores práticas de encaminhamento da comunidade médica e não médica.

## INTRODUCTION

Cryptorchidism, or undescended testis (UDT), is a prevalent congenital genitourinary condition, affecting approximately 4% of full-term male infants, 0.5 to 1% of adult males, and up to 30% of premature male infants.^1,2,3^

Testicular descent begins in the 10th week of intrauterine life and is typically completed by the 35th week. In 35–43% of cases, spontaneous descent occurs within the first three months, with the rate increasing to 70% by the end of the first year.^1,2,3,4,5,6^

Approximately 80% of all UDT cases are palpable. These can be located along the typical descent path, in the inguinal region, or in an ectopic position due to the abnormal descent during embryonic development, which occurs in about 15% of patients. Testis that can be easily moved downward but return to a suprascrotal position due to an overactive cremasteric reflex are classified as retractable testis.^6,7^ Among the 20% of non-palpable testes, 50–60% are located intra-abdominally. Bilaterality occurs in 10% of cases, and in 20% of these bilateral cases, at least one testis is non-palpable.^7^

The 2014 guideline by the American Urological Association (AUA) aimed to provide guidance to both primary care and specialized physicians, and non-physicians, on the management of UDT. Nine key recommendations were outlined for proper diagnosis, including a thorough medical history with details of the pregnancy, physical examination, and referral to a specialized service for orchidopexy.^6^

Since then, global protocols recommend that if spontaneous testicular descent does not occur by six months of age, surgery should be performed by 18 months to preserve fertility.^6-9^ This is because, at birth, 25% of patients have a reduced number of germ cells. Moreover, for every six-month delay in performing orchidopexy, the risk of testicular cancer increases by 6%, the need for assisted reproduction in the future rises by 5%, and the risk of infertility or inability to father children increases by 1%.^6-10^

The aim of the study was to assess the age at first consultation, the age at which surgical intervention was performed, and the duration of follow-up for patients with UDT at a tertiary care institution.

## METHOD

This is a retrospective review of all UDT patients who attended a tertiary care institution between 1992 and 2021. Ethical approval for the study was obtained from the institutional review board (CAAE 43517521.5.0000.5505).

The data collected included patients’ demographics and clinical characteristics, referral age, age at the time of orchidopexy, laterality, testis position before surgery, and the duration of postoperative follow-up. A descriptive analysis of each variable was conducted, excluding patients whose medical records lacked relevant data.

## RESULTS

Of the 350 patients studied, 72.6% had unilateral UDT, while 27.4% had bilateral UDT. Among the patients, 57.1% (200/350) had associated clinical conditions, which were more prevalent in those with bilateral UDT (65.8%; 25/38) and less common in patients with ectopic testis (40.0%; 2/5). Additionally, 8.0% (28/350) had differences in sex development (DSD), with the majority of cases occurring in patients with unilateral UDT (35.7% or 10/28 of those with DSD). Ultrasound was requested for 19.7% (69/350) of patients, particularly for those with unilateral UDT (37.7%).

Among the 309 patients (88.3%) who had data on their age at the first consultation, the average referral age was 47.3±41.6 months, with 72.5% of referrals occurring after 18 months. Excluding the 87 patients with retractile testis, the average referral age for the remaining 227 children was 51.0±45.3 months, with 70.9% being referred after 18 months (Table 1).

**Table 1:** Distribution of patients according to age at referral and type of undescended testis.

	0–6 month (%)s	6–12 months (%)	12–18 months (%)	>18 months (%)	Total (%)
Inguinal	19 (11.3)	17 (10.0)	13 (7.7)	120 (71.0)	169 (100)
Intra-abdominal	5 (9.3)	6 (11.0)	5 (9.3)	38 (70.4)	54 (100)
Retractile	3 (3.7)	9 (11.0)	7 (8.5)	63 (76.8)	82 (100)
Ectopic	0	1 (25.0)	0	3 (75.0)	4 (100)
Total	27 (8.7)	33 (10.7)	25 (8.1)	224 (72.5)	309 (100)

A total of 120 patients (34.3%) underwent surgical treatment, which included 131 orchidopexies and 10 orchiectomies. Eighteen patients underwent bilateral orchidopexy.

Orchidopexy was performed before 12 months in 12.8% of cases (based on data from 113 surgeries) and before 18 months, in 15.9%. The majority of surgeries (53.1%) were conducted after 60 months of age (Table 2). Given that most patients were older at the time of their first consultation, it can be inferred that the delay in referral is the primary reason for the delayed surgical treatment of UDT in patients at this institution.

**Table 2:** Distribution of patients by type of undescended testis and age at surgery.

	0–6 months (%)	6–12 months (%)	12–18 months (%)	18–60 months (%)	>60 months (%)	Total (%)
Inguinal	4 (6.1)	3 (4.5)	2 (3.0)	23 (34.9)	34 (51.5)	66 (100)
Intra-abdominal	3 (8.3)	3 (8.3)	1 (2.8)	9 (25.0)	20 (55.6)	36 (100)
Retractile	0	1 (12.5)	1 (12.5)	2 (25.0)	4 (50.0)	8 (100)
Ectopic	0	0	0	1 (33.3)	2 (66.7)	3 (100)
Total	7 (6.2)	7 (6.2)	4 (3.5)	35 (31.0)	60 (53.1)	113 (100)

Of the 120 patients who underwent orchidopexy or orchiectomy, only 78,3% (94/120) had information on the length of postoperative follow-up. Of these, 28,7% (27/94) were still being followed up during data collection and 19,1% (18/94) were discharged and 52,1% (49/94) were lost to follow-up. The average period of postoperative follow-up for patients who were discharged was 28.1 months and 11.9 months for patients who abandoned follow-up (Table 3). The characteristics of patients operated on for undescended testicles are shown in the Table 4.

**Table 3:** Follow-up of patients with undescended testis undergoing surgical treatment.

	Dropout (%)	Discharge (%)	Under medical supervision (%)	Total (%)
Inguinal	32 (56.1)	11 (19.3)	14 (24.6)	57 (100)
Intra-abdominal	13 (44.8)	4 (13.8)	12 (41.4)	29 (100)
Retractile	4 (50.0)	3 (37.5)	1 (12.5)	8 (100)
Total	49 (52.1)	18 (19.2)	27 (28.7)	94 (100)

**Table 4:** Characteristics of patients with undescended testis undergoing surgical treatment.

Characteristics	Number (%) or Mean±SD
Total patients	120
Total gonads	138
Unilateral disease	82 (68)
Undescended testis in the right side	75 (54)
Differences in sex development	21 (18)
Associated genitourinary conditions	68 (57)
Other associated conditions	87 (72)
Intraoperative site of testis	
Impalpable	36 (30)
Palpable	84 (70)
Retractile	8 (9)
Inguinal	72 (86)
Ectopic	4 (5)
Overall age of referral (months)	53.2±47.8
Overall age of orchiopexy (months)	72.8±51.8
Postoperative follow-up (months)	23.1±26.2

SD: standard deviation.

## DISCUSSION

Early diagnosis and referral of UDT are crucial to ensure that surgical intervention occurs at the optimal time, thereby reducing the risk of infertility. European, American, and British guidelines recommend that if spontaneous testicular descent has not occurred by six months of age, orchidopexy should be performed by 18 months to preserve fertility.^6-9^

In the present study, the referral age was notably late, with over 70.0% of patients being referred after 18 months, contributing to the older age observed at the time of orchidopexy. The delay in referral for UDT is a concern even in developed countries. Similar findings have been reported in other studies.^11,12,13,14,15,16,17,18,19,20^ Jiang et.al.^11^ found the average age at referral to be 44 months, with 64.0% after 18 months in 178 patients evaluated between 2017 and 2018 in Portland/USA. Another study, conducted in collaboration between West Virginia University and Johns Hopkins University in 2017, examined 131 patients from urban areas and 100 from rural areas, revealing an average referral age of 48.3 months and 59.6 months, respectively.^19^

Delay in referral has been linked to several factors, including a lack of awareness among healthcare professionals about the guidelines and benefits of early referral and surgery. Other contributing factors include failure to conduct a physical examination of the genitals at birth and during development, insufficient understanding of the physiology of UDT, follow-up loss, and inadequate parental education about the condition.^11^

According to the AUA guidelines, ultrasound and other imaging tests are contraindicated for diagnosing UDT due to their low sensitivity.^6,20^ However, these tests are still used in clinical practice. In our study, 19.5% of patients referred to the institution underwent imaging. The majority of these cases had UDT, which can typically be diagnosed through a thorough physical examination.^21^ Requesting such radiological exams may unnecessarily delay referrals and impact the timely indication for surgery. Studies indicate that ultrasound is also used in other countries despite being contraindicated. In an Australian study involving 145 patients with UDT, primary care confirmed the diagnosis by ultrasound in 87.6% of cases.^20^ However, only 49.6% of these diagnoses were actually confirmed as UDT by pediatric urologists. The study also found that patients over 8 years old were 5.2 times more likely to be misdiagnosed than those under this age. Furthermore, the ultrasound method showed an 81.0% false positive rate and a specificity of only 19.0%.^20^

In this study, DSD were found in 8.0% of the patients evaluated, with a higher prevalence in those with unilateral UDT. This highlights the importance of investigating DSD in patients with atypical genitalia, particularly those with one or two non-palpable gonads, as well as in cases of bilateral cryptorchidism, even when the genitalia appear typical.

According to the AUA guideline, orchidopexy should be performed by 18 months of age if there is neo spontaneous testicular descent by the sixth month of life.^6^ These timeframes are based on the impact UDT may have on fertility, as 25.0% of patients are born with a reduced number of germ cells. By the age of 8–11 years, the number of boys without germ cells in a testicular biopsy increases to approximately 40.0% in cases of bilateral UDT. Additionally, every 6-month delay in performing orchidopexy raises the risk of testicular cancer by 6.0%, the need for assisted reproduction in the future by 5.0%, and the likelihood of infertility or inability to father children by 1.0%.^6,10^

In this series, only 12.2% of surgeries were performed within the timeframe recommended by the guidelines, while 55.7% occurred after 5 years of age. In Germany, a study conducted between 2003 and 2016, involving patients from 16 different hospitals, found that only 4.0% of patients underwent orchidopexy in the first year of life, and 22.0% before completing their second year, prior to the revision of the national guideline.^12^ Even after the guideline was updated, between 2013 and 2016, only 8.0% of patients had orchidopexy in the first year, and 31.0% before the age of two.^12^ Additionally, a study conducted in collaboration between West Virginia University and Johns Hopkins University in 2017, involving 131 patients with UDT from urban areas and 100 from rural areas, showed an average age at surgical intervention of 53.8 months and 65.2 months, respectively. The surgery rate before 18 months was 29.0% for urban patients and 40.0% for rural patients.^19^ The study conducted in Bosnia and Herzegovina by Zvizdic et al.^16^ between 2008–2010 and 2015–2017 demonstrated that 64.8% of 324 patients underwent orchidopexy after 18 months, while only 28.8% underwent the procedure before 12 months, with the average age at surgery being 24 months.

Therefore, orchidopexy has been performed worldwide at older ages than recommended, despite the guidelines established since 2014.^11-19,22,23^ One key reason for this delay is the postponement in referring patients to pediatric surgical specialists. Public continuing education initiatives targeted at healthcare professionals in primary care settings, as well as information campaigns for caregivers, can improve referral timing.^24^ These measures could help avoid unnecessary delays, such as the indiscriminate use of scrotal ultrasound for diagnosing UDT.

Postoperative follow-up is crucial for detecting recurrence, which may require further surgical intervention, and for monitoring testicular health.^25,26^ In this study, 50.0% of patients were lost to follow-up, and another 20.0% were discharged prematurely. It is essential that guidelines related to fertility and cancer risk are clearly communicated to patients and their families, particularly during the transition to adult care. In this phase, the importance of self-examination and continued monitoring by a specialized team must be emphasized to ensure long-term health and well-being.

The limitation of this study was the lack of data on all the variables studied for the entire sample. However, it is evident that the average age at diagnosis and treatment of UDT in our institution was higher than the recommendations in American, European, and British guidelines. To address this issue, both medical and non-medical community initiatives should be implemented to improve the timely referral of patients with these clinical-surgical conditions.

In conclusion, the average age at diagnosis and treatment was higher than recommended by international guidelines, highlighting the need for improved referral practices within both the medical and non-medical communities.

## Data Availability

The database that originated the article is available with the corresponding author.
